# Impact of Long-Term Dienogest Therapy on Quality of Life in Asian Women with Endometriosis: the Prospective Non-Interventional Study ENVISIOeN

**DOI:** 10.1007/s43032-021-00787-w

**Published:** 2022-02-02

**Authors:** Kitirat Techatraisak, Andon Hestiantoro, Ruey Soon, Maria Jesusa Banal-Silao, Mee-Ran Kim, Seok Ju Seong, Syarief Thaufik Hidayat, Ling Cai, SoYoung Shin, Byung Seok Lee

**Affiliations:** 1 Department of Obstetrics and Gynecology, Gynecologic Endocrinology Unit, Faculty of Medicine, Siriraj Hospital, Mahidol University, Bangkok, Thailand; 2 Department of Obstetrics and Gynecology, Faculty of Medicine Universitas Indonesia, Cipto Mangunkusumo Hospital, Jakarta, Indonesia; 3 Department of Obstetrics and Gynecology, Sabah Women’s and Children’s Hospital, Kota Kinabalu, Malaysia; 4 Department of Obstetrics and Gynecology, St. Luke’s Medical Center Global City, University of the Philippines College of Medicine, Philippine General Hospital, Manila, Philippines; 5 Department of Obstetrics & Gynecology, Seoul St. Mary’s Hospital, College of Medicine, The Catholic University of Korea, Seoul, Republic of Korea; 6 Department of Obstetrics and Gynecology, CHA Gangnam Medical Center, CHA University, Seoul, Republic of Korea; 7 Department of Obstetrics and Gynecology, Hermina Pandanaran Hospital, Semarang, Indonesia; 8 Bayer Pharma AG, Wuppertal, Germany; 9 Former employee of Bayer AG, Seoul, Republic of Korea; 10 Division of Gynecologic Endocrinology and Infertility, Department of Obstetrics and Gynecology, Severance Hospital, Yonsei University, Seoul, Republic of Korea; 11 Department of Obstetrics and Gynecology, Severance Hospital, Yonsei University, 50-1 Yonsei-ro, Seodaemun-gu, 120-752 Seoul, Republic of Korea

**Keywords:** Endometriosis, Health-related quality of life, Pain, Clinical diagnosis

## Abstract

Several clinical trials in women with endometriosis demonstrated that dienogest reduces endometrial lesions and improves health-related quality of life (HRQoL). To assess HRQoL in dienogest-treated patients in real-world setting, we conducted a prospective, non-interventional study in 6 Asian countries. Women aged ≥18 years with clinical or surgical diagnosis of endometriosis, presence of endometriosis-associated pelvic pain (EAPP) and initiating dienogest therapy were enrolled. The primary objective was to evaluate HRQoL using the Endometriosis Health Profile-30 (EHP-30) questionnaire. The secondary objectives included analysis of EAPP, satisfaction with dienogest, endometriosis symptoms and bleeding patterns. 887 patients started dienogest therapy. Scores for all EHP-30 scales improved with the largest mean changes at month 6 and 24 in scale pain (−28.9 ± 27.5 and − 34 ± 28.4) and control and powerlessness (−23.7 ± 28.2 and − 28.5 ± 26.2). Mean EAPP score change was −4.6 ± 3.0 for both month 6 and 24 assessments. EAPP decrease was similar in surgically and only clinically diagnosed patients. From baseline to month 24, rates of normal bleeding decreased (from 85.8% to 17.5%) while rates of amenorrhea increased (from 3.5% to 70.8%). Majority of patients and physicians were satisfied with dienogest. Over 80% of patients reported symptoms improvement. 39.9% of patients had drug-related treatment-emergent adverse events, including vaginal hemorrhage (10.4%), metrorrhagia (7.3%) and amenorrhea (6.4%). In conclusion, dienogest improves HRQoL and EAPP in the real-world setting in women with either clinical or surgical diagnosis of endometriosis. Dienogest might be a promising first-line treatment option for the long-term management of debilitating endometriosis-associated symptoms.

NCT02425462, 24 April 2015.

## Introduction

Endometriosis affects approximately 10% of women in the reproductive age, with a prevalence of up to 50% in infertile women [1, 2]. The prevalence of endometriosis varies by race and ethnicity with Asian women (particularly in East Asian countries) more likely to be affected by this disease than Caucasians [1, 3–5]. The most common clinical signs of endometriosis are menstrual irregularities, chronic pelvic pain, dysmenorrhea, dyspareunia and infertility [6]. Symptoms of endometriosis often affect psychological and social functioning of patients. For this reason, endometriosis is considered as a disabling condition that may significantly reduce health-related quality of life (HRQoL). Previous studies demonstrated that endometriosis has a negative impact on social relationships, sexuality, mental health, and work productivity [7–10]. Furthermore, poor HRQoL is observed particularly in patients suffering from a more severe pelvic pain [10–12]. Importantly, hormonal therapy and surgical treatment may improve HRQoL and reduce pelvic pain in patients with endometriosis [13].

Dienogest is an oral progestin approved for endometriosis treatment in 157 countries, including 15 countries in Asia [7]. Dienogest is an attractive option for prolonged treatment due to a moderate suppression of estrogen levels and a low androgenic, mineralocorticoid, or glucocorticoid activity [14]. Long-term studies in Europe and Japan demonstrated that dienogest is efficacious in reducing pelvic pain, well tolerated and has a good safety profile [15–17]. Although several clinical trials demonstrated improvements in HRQoL following the dienogest therapy, the real-world evidence is scarce [18–24]. Our interim analysis of non-interventional study ENVISIOeN demonstrated an improved HRQoL and reduced endometriosis-associated pelvic pain (EAPP) after 6 months of dienogest therapy in clinically or surgically diagnosed Asian women with endometriosis [25]. In this manuscript, we report the final analysis of HRQoL, EAPP and safety in patients receiving dienogest for up to 24 months within the ENVISIOeN study.

## Methods

### Study Design

The ENVISIOeN study (NCT02425462) was a prospective, multicenter, international, noninterventional cohort study performed in 6 Asian countries at 36 sites (Republic of Korea: 12 sites, Indonesia: 10 sites, Thailand: 5 sites, Malaysia: 4 sites, Philippines: 3 sites, Singapore: 2 sites). The study was conducted in accordance with the Declaration of Helsinki and in compliance with Good Clinical Practice.

The primary objective of the study was to determine the change in pain dimension of Endometriosis Health Profile-30 (EHP-30) after 6-month therapy with dienogest. The secondary objectives included change in EHP-30 scores at month 6 and 24, efficacy of dienogest in reducing EAPP at month 6, 12 and 24, patients’ and clinicians’ satisfaction with dienogest, assessment of endometriosis symptoms and bleeding pattern, and rates of dienogest discontinuation and repeated surgery. Data were collected during study visits at basline, and at months 1, 3, 6, 12, and 24 after start of dienogest therapy; all study visits occurred on site and within the routine clinical practice.

### Patients

Asian women aged ≥18 years with clinical diagnosis (by suggestive symptoms and positive finding of chocolate cyst on imaging) or surgical diagnosis of endometriosis and presence of EAPP were eligible to participate in the study. In this study, EAPP was defined as pain at menstruation, chronic pelvic pain irrelevant to menstruation, and/or dyspareunia. Further inclusion criteria were physician’s independent decision to newly prescribe dienogest and written informed consent provided by patient. Exclusion criteria were contraindication for dienogest use as per the summary of product characteristics (active venous thromboembolic disorder, arterial and cardiovascular disease, diabetes mellitus with vascular involvement, severe hepatic disease, liver tumors, known or suspected sex hormone-dependent malignancies, undiagnosed vaginal bleeding and hypersensitivity to the active substance or to any of the excipients) and participation in an investigational program with interventions outside of routine clinical practice. Patient were treated according to the respective national guidelines and in line with the American Society for Reproductive Medicine and/or European Society of Human Reproduction and Embryology guidelines.

### Assessments

Enrolled patients were observed over the 24-month period, irrespective of whether they remained or discontinued dienogest, unless lost to follow up. Patients filled out the EHP-30 questionnaire at baseline and at month 6 and month 24. EHP-30 consists of the core instrument scales addressing pain, control and powerlessness, social support, emotional well-being, and self-image [26–28]. Additionally, supplementary module scales covering areas of work, relationship with children, sexual relationship, treatment, and infertility were used. Items for each scale were summed and transformed on a range from 0 (best possible HRQoL) to 100 (worst possible HRQoL).

Patients evaluated EAPP using the numeric rating scale with scores ranging from 0 (no pain) to 10 (unbearable pain) at baseline and after 6 and 24 months of therapy with a 4-week recall period.

Bleeding profile was analyzed across the following categories: normal bleeding (regular bleeding with normal flow and duration), irregular bleeding cycle (intermenstrual interval < 21 or > 35 days), amenorrhea (no menstruation during last 90 days), and intermenstrual spotting/bleeding (irregular episodes of bleeding, often light and short, occurring between otherwise fairly normal menstrual periods).

Further assessments included the rate and reason for dienogest discontinuation, patients’ and physicians’ satisfaction with treatment, and pain recurrence and repeated surgery rates.

Treatment-emergent adverse events (TEAE) and serious adverse events (SAE) were documented at each study visit.

### Statistical Analysis

Analysis of efficacy was performed on the efficacy analysis set (EFF) that included all patients with evaluable primary outcome, i.e., EHP-30 questionnaires filled out at baseline and between week 12 and 36 after start of treatment (6-month visit) by patients who continued dienogest therapy. Measurements after dienogest discontinuation were excluded. Safety analyses were performed on the full analysis set (FAS) that comprised of all patients with at least one dose of dienogest.

Data were analyzed using SAS release 9.4 (SAS Institute, Cary, NC, USA) and described by visit (baseline, 6- and 24-month visit) and by change from baseline. Continuous variables were summarized by mean (± standard deviation), median, minimum, maximum; and categorical variables as a number and percentage. 95% confidence intervals (CI) were provided for changes in EHP-30 scores. Missing data were not imputed. Incidence of TEAE and SAE was presented as the Medical Dictionary for Regulatory Activities (MedDRA) preferred terms by system organ class.

## Results

### Patient Disposition and Characteristics

**Fig. 1:**
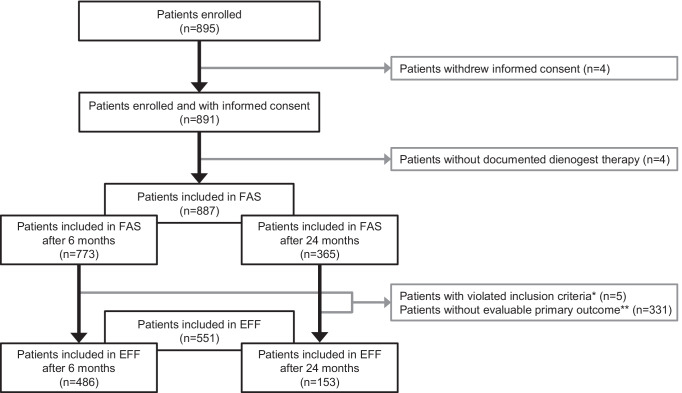
CONSORT diagram. *One patient violated inclusion criteria: clinical or surgical diagnosis of endometriosis and endometriosis-associated pelvic pain, and four patients violated inclusion criterion decision taken by the physician to newly prescribe dienogest. **EHP-30 pain score was not evaluable at baseline and/or at study visit at month 6. EFF, efficacy analysis set; FAS, full analysis set

From April 2015 to August 2016, 895 patients at 36 sites were enrolled in the study (Fig. 1). 887 patients were included in FAS; 551 patients were included in EFF set. The mean duration of newly prescribed dienogest treatment in EFF set was 16.9 ± 7.6 months and the main reasons for end of observation were regular end of study (64.4%), lost to follow-up (23.1%) and relief of symptoms (6.7%). At month 6, the majority of patients continued treatment with dienogest (85.3%); 13.3% of patients discontinued the therapy, most often due to relief of symptoms (38.4%), adverse event (AE, 35.6%) or wish to conceive (9.6%). At month 24, dienogest therapy was continued by 44.2% of patients whereas 28.8% discontinued the dienogest, most frequently due to relief of symptoms (53.6%), physician decision (17.9%) or AE (14.3%).

536 patients (60.4%) received endometriosis treatment prior to study start (Table 1); 454 (84.7%) among them underwent prior surgery and 186 women (34.7%) had previous hormonal treatment, most often gonadotropin-releasing hormone agonist (55.4%), followed by progestin plus estrogen-progestin combination (29.6%) and progestin alone (19.9%); 3.2% of patients received dienogest. Pain recurred in 28.2% of women with surgery (within 17.6 ± 21.2 months), and in 56.5% of patients with prior hormonal treatment (within 8.0 ± 11.9 months). Pain medication was given to 18.3% of patients (*n* = 98/536); mean time to pain recurrence was 9.7 ± 33.5 days.

**Table 1:** Baseline characteristics

Parameter		
Demography	N	years, mean ± SD
Age at registration	887	34.4 ± 7.6
History of endometriosis	N	%
Onset of first symptomsa		
<1 year ago	407	45.9
Between 1 and 5 years ago	273	30.8
>5 years ago	206	23.2
Missing	1	0.1
Most common symptoms of endometriosisb		
Dysmenorrhea	720	81.2
Chronic pelvic pain	304	34.3
Dyspareunia	55	6.2
Subfertility	44	5.0
Time point of first diagnosisa		
<1 year ago	651	73.4
Between 1 and 5 years ago	145	16.4
>5 years ago	91	10.3
Method of diagnosisb		
Surgical diagnosis	621	70.0
Clinical diagnosis	560	63.1
Endometriosis lesions (n = 777 evaluable patients)		
Single	425	54.7
Multi	352	45.3
Endometriosis localization (n = 737 evaluable patients)b		
Ovary	650	88.2
Pelvic organ	287	38.9
Extra pelvic	21	2.9
Prior endometriosis treatment	N	%
Patients without prior treatment	351	39.6
Patients with prior treatment	536	60.4
Type of treatment (*n* = 536)	N	%
Surgery	454	84.7
Hormonal treatment	186	34.7
Pain therapy	98	18.3
rASRM stage of endometriosis (n = 471)^b,c^	N	%
Stage I (minimal)	17	3.7
Stage II (mild)	25	5.5
Stage III (moderate)	92	20.3
Stage IV (severe)	130	28.6
Unknown	207	45.6
Most common previous diseases (*n* = 145)^b^	N	%
Uterine leiomyoma	71	49.0
Endometrial polyp	36	24.8
Ovarian cysts	26	17.9
Pelvic inflammatory disease	20	13.8
Most common concomitant diseases (*n* = 154)^b^	N	%
Adenomyosis	51	33.1
Uterine leiomyoma	42	27.3
Anemia	18	11.7

^a^Partially missing dates were imputed by the earliest possible time point: In case that only the day was missing, the date was imputed as the first day of the month. In case that the day and the month were missing, i.e., only the year was available, the day and month was imputed by January 1st

^b^Multiple answers possible

^c^rASRM was assessed for each individual surgery (*n* = 471 surgeries) in 454 patients with a prior surgical treatment for endometriosis. rASRM, revised American Society for Reproductive Medicine score

### EHP-30 Scores

**Fig. 2:**
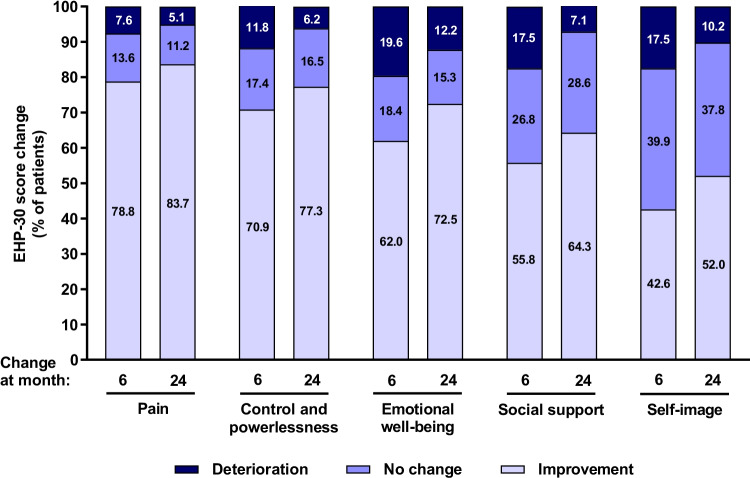
Changes in core EHP-30 scores from baseline to month 6 and to month 24. Results demonstrate proportions of patients with deterioration, no change and improvement defined as >0, 0 and < 0 difference in EHP-30 scores between baseline and visit at month 6 and 24. Data were analyzed in patients with evaluable score changes after 6 (n = 486, scales pain, social support, self-image; n = 484, scales control and powerlessness and emotional well-being) and after 24 months of therapy (n = 98, scales pain, emotional well-being, social support and self-image; *n* = 97, scale control and powerlessness). EHP-30, Endometriosis Health Profile-30 questionnaire

For 6-month and 24-month assessments, EHP-30 core instrument questionnaires were filled out on average at 178 ± 22.5 days (*n* = 486) and 713.2 ± 30.9 days (n = 98) after baseline visit, respectively, and EHP-30 modular instrument questionnaires were filled out on average 178 ± 22.2 days (*n* = 482) and 713.7 ± 30.4 days (*n* = 100) after baseline visit, respectively. Scores for all EHP-30 core and modular instrument scales improved during the first 6 months of dienogest therapy and continued to improve until month 24 (Tables 2 and 3, respectively). Percentage of patients with improvement in EHP-30 scores was highest for scales Pain, and Control and powerlessness (Fig. 2).

**Table 2:** EHP-30 core scores and changes from baseline

Dimension	N	Mean	SD	Median	Min; max	95%CI	Nmiss
Pain							
Baseline	551	37.5	28.1	38.6	0; 100	–	0
Month 6	486	8.4	13.5	0	0; 65.9	–	0
Month 24	98	3.9	8.4	0	0; 38.6	–	55
Change from baseline at month 6	486	−28.9	27.5	−25	−100; 36.4	−31.35; −26.45	0
Change from baseline at month 24	98	−34	28.4	−30.7	−100; 29.6	−39.66; −28.29	55
Control and powerlessness							
Baseline	550	35.3	28.1	33.3	0; 100	–	1
Month 6	485	11.4	16.2	4.2	0; 87.5	–	1
Month 24	98	5.7	9.9	0	0; 58.3	–	55
Change from baseline at month 6	484	−23.7	28.2	−18.8	−100; 58.3	−26.23; −21.19	2
Change from baseline at month 24	97	−28.5	26.2	−29.2	−100; 25	−33.81; −23.23	56
Emotional well-being							
Baseline	549	32.3	25.9	29.2	0; 100	–	2
Month 6	485	17.3	20.3	8.3	0; 91.7	–	1
Month 24	98	9.8	16.8	0	0; 75	–	55
Change from baseline at month 6	484	−15	26.7	−10.4	−100; 79.2	−17.35; −12.57	2
Change from baseline at month 24	98	−20.6	22.8	−20.8	−70.8; 33.3	−25.19; −16.05	55
Social support							
Baseline	550	27.7	25.8	25	0; 100	–	1
Month 6	486	14.4	19.8	6.3	0; 87.5	–	0
Month 24	98	8.4	18	0	0; 100	–	55
Change from baseline at month 6	487	−13.2	27	−6.3	−100; 87.5	−15.56; −10.75	0
Change from baseline at month 24	98	−17.9	24.3	−12.5	−87.5; 43.8	−22.79; −13.05	55
Self-image							
Baseline	550	20.6	24.9	8.3	0; 100	–	1
Month 6	486	11.7	18.9	0	0; 91.7	–	0
Month 24	98	7.1	17.3	0	0; 100	–	55
Change from baseline at month 6	486	−8.4	26.4	0	−100; 91.7	−10.76; −6.05	0
Change from baseline at month 24	98	−13.9	23.2	−8.3	−83.3; 50	−18.52; −9.20	55

**Table 3:** EHP-30 modular scores and changes from baseline

Dimension	N	Mean	SD	Median	Min; max	95%CI	Nmiss	N/A*
Work								
Baseline	475	30.4	27.1	30	0; 100	–	13	63
Month 6	431	7.5	13.6	0	0; 80	–	7	46
Month 24	86	2.8	8.8	0	0; 60	–	54	14
Change from baseline at month 6	395	−22.4	25.4	−15	−90; 40	−24.88; −19.86	89	
Change from baseline at month 24	81	−25.4	25	−20	−90; 35	−30.89; −19.85	73	
Relationship with children								
Baseline	189	25.5	30.4	0	0; 100	–	27	335
Month 6	188	6.7	14.7	0	0; 75	–	29	267
Month 24	41	2.4	7	0	0; 25	–	17	96
Change from baseline at month 6	140	−18.5	29.4	0	−100; 50	−23.39; −13.57	344	
Change from baseline at month 24	30	−25.8	28.6	−25	−100; 12.5	−36.51; −15.15	124	
Sexual intercourse								
Baseline	353	28.1	26.8	25	0; 100	–	79	119
Month 6	303	15	20.5	5	0; 95	–	36	145
Month 24	66	11.1	18.3	0	0; 75	–	62	26
Change from baseline at month 6	258	−13.1	26.4	−5	−90; 75	−16.29; −9.82	226	
Change from baseline at month 24	52	−17.8	27.5	−10	−75; 40	−25.45; −10.17	102	
Treatment								
Baseline	383	19.9	22.7	16.7	0; 100	–	31	137
Month 6	447	11.2	16.3	0	0; 83.3	–	6	31
Month 24	92	6.9	13	0	0; 75	–	54	8
Change from baseline at month 6	322	−7.2	22.4	0	−91.7; 50	−10.43; −3.96	162	
Change from baseline at month 24	70	−11.6	25.2	0	−91.7; 50	−17.56; −5.53	84	
Infertility								
Baseline	317	29	28.8	25	0; 100	–	36	198
Month 6	308	20.2	24.1	12.5	0; 100	–	23	153
Month 24	59	14.2	18.3	6.3	0; 81.3	–	57	38
Change from baseline at month 6	235	−8.9	21.9	0	−100; 56.3	−11.69; −6.07	249	
Change from baseline at month 24	47	−11.4	19.4	−12.5	−75; 31.3	−17.14; −5.73	107	

*Not applicable to the respective issue during the last 4 weeks

### EAPP

**Fig. 3:**
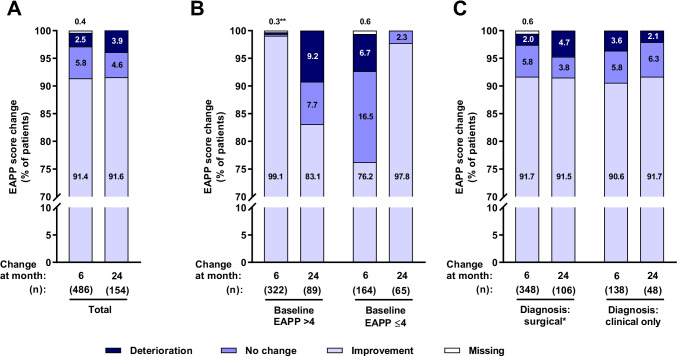
Changes in EAPP from baseline to month 6 and to month 24 among all patients included in efficacy analysis set (**a**) and according to type of diagnosis (**b**) and baseline EAPP severity (**c**). Data shown are proportions of patients with deterioration, no change and improvement defined as > 0, 0 and < 0 difference in EAPP scores between baseline and visits at months 6 and 24. *Including patients with surgical diagnosis only and surgical and clinical diagnosis. **0.3% of patients each had no change, improvement or missing data on change of EAPP. EAPP, endometriosis-associated pelvic pain

EAPP assessment at month 6 and month 24 was performed on average 175.6 ± 22.6 days (*n* = 484) and 701.1 ± 41 days (*n* = 154) after baseline visit. EAPP score improved during the first 6 months of therapy and remained stable thereafter (Table 4). Patients with baseline EAPP >4 had a greater mean EAPP score change than those with baseline EAPP ≤4 during therapy. Patients not taking rescue medication (defined as any medication for pain management used while on treatment with dienogest) showed a tendency towards an improved EAPP compared to those taking the rescue medication (note that only a few patients were included in the latter category). Type of diagnosis (surgical vs only clinical) and prior surgical or hormonal treatment had no impact on efficacy of dienogest to alleviate EAPP (Table 4). More than 90% of patients had an improvement in EAPP at both post-baseline study visits (Fig. 3). Amelioration of EAPP was more often experienced at month 6 by women with a higher baseline EAPP severity (>4), and at month 24 by those with a lower baseline EAPP (≤4).

**Table 4:** EAPP scores and changes from baseline

Parameter		N	Mean	SD	Median	Min, max	Nmiss
Total	Baseline	551	5.6	2.8	6	0; 10	0
	Month 6	484	1	1.4	0	0; 7	2
	Month 24	154	0.7	1.3	0	0; 9	0
	Change from baseline at month 6	484	−4.6	3	−5	−10; 5	2
	Change from baseline at month 24	154	−4.6	3	−5	−10; 3	0
Surgical diagnosis*	Baseline	396	5.3	2.8	6	0; 10	0
	Month 6	346	1	1.4	0	0; 7	2
	Month 24	106	0.8	1.3	0	0; 5	0
	Change from baseline at month 6	346	−4.3	2.8	−4	−10; 4	2
	Change from baseline at month 24	106	−4.5	3	−5	−10; 3	0
Clinical diagnosis only	Baseline	155	6.3	2.7	7	0; 10	0
	Month 6	138	1	1.5	0	0; 6	0
	Month 24	48	0.6	1.4	0	0; 9	0
	Change from baseline at month 6	138	−5.3	3.2	−6	−10; 5	0
	Change from baseline at month 24	48	−4.8	3.1	−5	−10; 2	0
Baseline severity of EAPP ≤4	Baseline	185	2.3	1.2	2	0; 4	0
	Month 6	163	0.8	1.3	0	0; 6	1
	Month 24	65	0.6	1.1	0	0; 5	0
	Change from baseline at month 6	163	−1.5	1.7	−1	−4; 5	1
	Change from baseline at month 24	65	−1.8	1.6	−2	−4; 3	0
Baseline severity of EAPP >4	Baseline	366	7.3	1.6	7	5; 10	0
	Month 6	321	1	1.5	0	0; 7	1
	Month 24	89	0.8	1.5	0	0; 9	0
	Change from baseline at month 6	321	−6.2	2.1	−6	−10; 1	1
	Change from baseline at month 24	89	−6.6	2	−7	−10; 0	0
Previous surgical or hormonal treatment	Baseline	329	5.3	2.7	6	0; 10	0
	Month 6	283	0.9	1.4	0	0; 7	2
	Month 24	93	0.6	1.1	0	0; 5	0
	Change from baseline at month 6	283	−4.4	2.8	−5	−10; 3	2
	Change from baseline at month 24	93	−4.3	2.7	−4	−9; 1	0
No previous surgical or hormonal treatment	Baseline	222	6	2.8	6	0; 10	0
	Month 6	201	1	1.4	0	0; 6	0
	Month 24	61	0.9	1.6	0	0; 9	0
	Change from baseline at month 6	201	−4.9	3.2	−5	−10; 5	0
	Change from baseline at month 24	61	−5	3.5	−5	−10; 3	0
Use of rescue medication	Baseline	55	6.7	2.6	8	0; 10	0
	Month 6	13	2.9	1.8	3	0; 5	0
	Month 24	3	2.3	1.2	3	1; 3	0
	Change from baseline at month 6	13	−3.2	3.4	−3	−10; 4	0
	Change from baseline at month 24	3	−2.7	5.1	−4	−7; 3	0
No use of rescue medication	Baseline	496	5.5	2.8	6	0; 10	0
	Month 6	471	0.9	1.4	0	0; 7	0
	Month 24	151	0.7	1.3	0	0; 9	0
	Change from baseline at month 6	471	−4.7	3	−5	−10; 5	0
	Change from baseline at month 24	151	−4.6	3	−5	−10; 3	0

*Includes patients, who only had surgical diagnosis as well as surgical + clinical diagnosis. EAPP, endometriosis-associated pelvic pain

Pain recurrence rate in EFF set was approximately three times higher in patients discontinuing (8.3%, *n* = 8/97) than among women continuing dienogest treatment for up to 24 months (2.7%, *n* = 4/146). Median time from dienogest discontinuation to pain recurrence was 22.2 months (Q1–Q3: 22.2–22.2).

### Bleeding Pattern

**Fig. 4:**
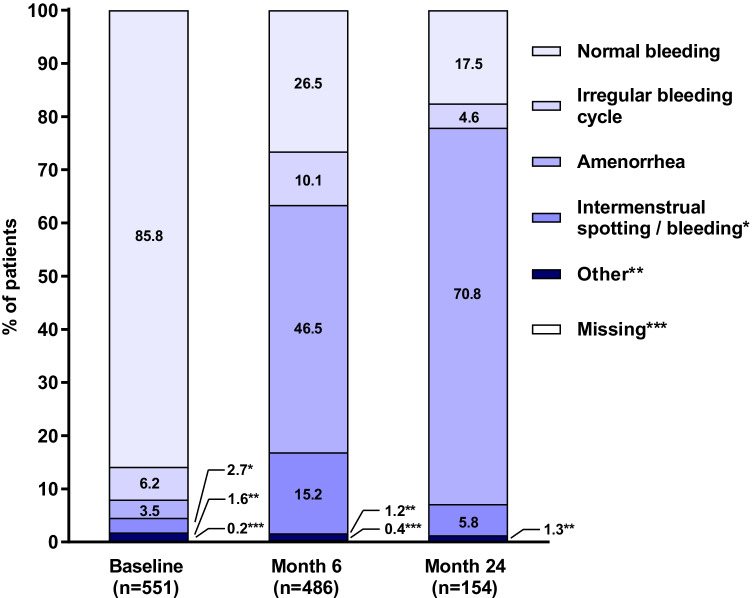
Bleeding patterns at the baseline, month 6 and month 24. The bleeding patterns were defined as follows: normal bleeding: regular bleeding with normal flow and duration; irregular bleeding cycle: bleeding cycle less than 21 days or more than 35 days; amenorrhea: no menstruation during last 90 days; intermenstrual spotting/bleeding: irregular episodes of bleeding, often light and short, occurring between otherwise fairly normal menstrual periods

Share of patients with normal bleeding decreased while the rate of amenorrhea increased during the 24-month therapy period (Fig. 4). The percentage of patients with irregular bleeding and intermenstrual spotting/bleeding increased during the first 6 months of therapy and then returned to baseline levels at month 24.

### Evaluation of Satisfaction and Symptoms

66.6% of patients (*n* = 515/773 of FAS) and 67.7% (*n* = 523/773) of physicians at month 6 and 52.3% (*n* = 191/365) of both patients and physicians at month 24 were very satisfied or somewhat satisfied with dienogest. At month 6 and month 24, only 4.5% and 2.5% of patients, and 2.1% and 1.9% of physicians were dissatisfied with treatment. 83.7% (*n* = 407/486 of EFF) and 87.0% of patients (*n* = 134/154) reported improved of symptoms at month 6 and month 24, respectively. Only 1.0% and 1.3% of patients reported that their symptoms worsened at month 6 and month 24, respectively. The proportion of patients receiving another or no treatment increased from 1.1% at month 6 to 27.1% at month 24. Surgery was repeated in two out of 273 patients that received a surgery prior to dienogest, including one patient (1.3%) among the 77 patients who stopped dienogest and one patient (0.5%) in the group of 196 patients that continued therapy until month 24.

### Safety Analysis

TEAEs, predominantly of mild-to-moderate intensity, were reported by 45.9% of patients (*n* = 407/887). TEAEs occurring in at least 1% of patients are presented in Table 5.

**Table 5:** Treatment-emergent adverse events observed in at least 1% of patients

TEAE (MedDRA PT)	N	%
Any TEAE	407	45.9
Vaginal hemorrhage	95	10.7
Metrorrhagia	75	8.5
Amenorrhea	65	7.3
Weight increased	41	4.6
Menstruation irregular	39	4.4
Headache	33	3.7
Acne	23	2.6
Alopecia	23	2.6
Depression	16	1.8
Menorrhagia	16	1.8
Insomnia	13	1.5
Abdominal pain	12	1.4
Dizziness	12	1.4
Uterine hemorrhage	10	1.1
Breast tenderness	9	1

*MedDRA* Medical Dictionary for Regulatory Activities, *PT* preferred term, *TEAE* Treatment-emergent adverse event

A total of 616 drug-related TEAEs were documented in 39.9% of patients (*n* = 354/887), most often vaginal hemorrhage (defined as any bloody discharge outside of normal menstrual cycle, including prolonged or irregular bleeding/spotting, in 10.4% of patients), metrorrhagia (defined as abnormal bleeding between regular menstrual periods, in 7.3%) and amenorrhea (6.4%). 63 patients (7.1%) discontinued dienogest therapy due to drug-related TEAEs. Abnormal uterine bleeding (MedDRA preferred terms: metrorrhagia, menorrhagia, vaginal or uterine hemorrhage) was the most prominent reason for discontinuation (2.0%, *n* = 18/887).

Eleven SAEs were reported in nine patients (Table 6). Anemia and menorrhagia (two events each) were the most common SAEs. Ten events were classified as serious because they required hospitalization and one case of anemia was an important medical event. All SAEs were recovered or resolved.

**Table 6:** All documented serious adverse events

SAE (MedDRA PT)	N	%
Any SAE	9	1
Anemia	2	0.2
Menorrhagia	2	0.2
Vomiting*	1	0.1
Asthenia*	1	0.1
Bartholin’s abscess	1	0.1
Leptospirosis	1	0.1
Peritonitis	1	0.1
Dysmenorrhea*	1	0.1
Ovarian cyst	1	0.1

*Drug-related SAE. *MedDRA* Medical Dictionary for Regulatory Activities, *PT* preferred term, *SAE* serious adverse event

## Discussion

Almost half of women with endometriosis are dissatisfied with their medical treatment [29, 30]. This indicates that there is an unmet need for therapeutic approaches effective in alleviating disease symptoms and maintaining good HRQoL. Although surgery is efficacious in alleviating endometriosis symptoms and improving HRQoL, 40% to 50% of patients have symptom recurrence, with 47% needing reoperation [31–34]. For these reasons, several guidelines recommend empirical long-term medicinal treatment and taking into account specific needs and expectations of the patient [2, 7, 35–37]. In our study, we found that dienogest improved scores for all EHP-30 scales already at month 6 of treatment, with continuous improvement until month 24. Similarly, continuous improvements in EHP-30 scores were noted in adolescents over the 12-month dienogest therapy [22]. In long-term, dienogest improved all EHP-30 core scores in patients with rectosigmoid endometriosis treated for 36 months [38]. Furthermore, 6-month treatment with dienogest increased HRQoL in patients who had a pain persistence and were unsatisfied with norethisterone acetate therapy [39]. In addition to EHP-30 questionnaire, Caruso and colleagues reported an improved HRQoL as assessed by SF-36 questionnaire in patients treated with dienogest for 6 months [20]. That study also demonstrated an increased sexual function and reduced sexual distress following the therapy. Collectively, these data indicate that dienogest rapidly improves HRQoL and is effective in a long-term. Moreover, EAPP scores improved during 6 months of dienogest therapy, and this effect was maintained over the entire observation period. Thus, our results confirm data on EAPP from other observational studies investigating long-term dienogest therapy. For example, in the study by Park et al., EAPP decreased by 33.5 mm on visual analog scale during 12 months of therapy [40] while Römer reported a decrease in EAPP of 50 mm after 60 months of treatment [41]. Furthermore, in a large open-label extension study following a 12-week placebo-controlled trial, EAPP decrease amounted to 43.2 mm at week 65 [15]. Rate of patients with EAPP reduction in our study (over 91% of patients) was slightly lower than previously reported in clinical trials (96.7% to 97.1%) [40, 42]. Furthermore, patients with baseline EAPP score > 4 had a more pronounced pain reduction than those with baseline EAPP score ≤ 4. Interestingly, percentage of patients with improvement in pain scores decreased from month 6 to month 24 among those with baseline EAPP score > 4 and increased in those with score ≤ 4. On the one hand, these data indicate that a longer duration of dienogest therapy is required to alleviate EAPP in patients with low baseline EAPP score. On the other hand, some patients with a higher initial EAPP severity might require rescue medication during therapy. The latter notion is supported by the fact that only a few patients had documented use of rescue medication. A lower EAPP improvement in that group compared with patients not taking rescue medication stresses the need for appropriate pain management in patients with severe EAPP. Furthermore, prior treatment (surgery or hormonal therapy) and type of diagnosis (surgical or clinical) had no impact on magnitude of EAPP improvement. Similarly, no difference in EAPP reduction between patients with or without surgery was reported in the study by Römer et al. [41]. This suggests that dienogest is efficacious in reducing EAPP in both first- and later lines of therapy and it is an optimal choice when access to surgical diagnostic techniques like laparoscopy is limited. Several guidelines now emphasize the value of non-invasive clinical diagnosis based on clinical symptoms and patient’s history, especially in low-resource setting [2, 35, 37]. Collectively, our results support a new paradigm that the diagnosis of endometriosis does not always require histological data, but with appropriate clinical approaches and empirical therapy, we can induce long-term improvements in quality of life in [2, 7, 35–37]. This approach would be even better if we succeeded in having non-invasive endometriosis diagnostic markers [43–45].

Reduction in severity of EAPP potentially contributed to the fact that 66.6% of patients after month 6 and 52.3% after month 24 were satisfied with dienogest therapy. Similar or even higher rates of satisfaction with dienogest therapy were reported by European patients [22, 41, 46]. Considering that 40% to 45.5% of women with endometriosis are dissatisfied with their current treatment, data accumulated thus far suggest that dienogest largely meets expectations of patients regarding their treatment [29, 30].

Analysis of TEAE revealed that rate of patients with depression was similar or lower than previously reported (1.8% vs 2.0% to 82.9%, [18, 22, 38, 41, 42]). Furthermore, fewer patients reported headache than in other studies (3.7% vs 9.0% to 12.5%, [18, 22, 41, 42]). Rates of weight gain, acne and alopecia were slightly lower or similar as previously reported [22, 38, 42]. Only 1% of patients had SAE, mostly anemia and menorrhagia. 39.9% of patients had drug-related TEAE while previous studies reported either higher or lower rates of drug-related AE (15.3% to 100%, [15, 22, 46, 47]). It should be noted however, that dienogest was used at various doses in these studies and most of reported drug-related AE were non-serious. Yu et al. reported lower rates for dienogest-related vaginal hemorrhage and amenorrhea (1.8%, both, vs 10.4% and 6.4%, respectively, in our study [46]). Conversely, Harada et al. demonstrated a higher frequency of genital bleeding (95%, [47]). Overall, the share of patients with normal bleeding profile decreased while proportion of patients with amenorrhea increased during the study which is in line with current evidence on dienogest profile [7, 15, 17, 18, 42, 48]. Therefore, physicians should inform patients about potential abnormal uterine bleedings which could occur during long-term treatment with progestins.

The key strengths of our study are the large sample size spanning 6 Asian countries and the long duration of follow-up. Additionally, by using disease-specific patient-reported outcome measures, we captured the patients’ perspective on changes in HRQoL during therapy. Furthermore, broad inclusion criteria ensured collection of real-world evidence in unselected population of patients treated with dienogest. Our study has also several limitations. The number of patients included in the efficacy analysis decreased from month 6 to month 24, therefore, results from the latter time-point may not be representative to all investigated patients. Furthermore, due to international setting of this study, differences between the healthcare systems could affect the diagnosis or type of rescue treatment of endometriosis. Finally, the observational nature of this study precluded the comparison of efficacy and safety between dienogest and other medicines for endometriosis.

## Conclusion

The results of the ENVISIOeN study indicate that dienogest improves patient-reported HRQoL and EAPP in the real-life setting in Asian women with endometriosis diagnosed either clinically or surgically. Given that observed safety profile was consistent with the previous results and that satisfaction with the treatment was high, dienogest might be a promising first-line treatment option for the long-term management of only clinically diagnosed patients with debilitating endometriosis-associated symptoms.
